# 38 Utilizing an Implementation Science Framework to Design a Burn Resuscitation Bundle in a Resource-limited Setting

**DOI:** 10.1093/jbcr/irac012.041

**Published:** 2022-03-23

**Authors:** Kajal Mehta, Joohee Lee, Aldina Mesic, Manish K Yadav, Raslina Shrestha, Kiran Poudel, Prakriti Gyawali, Tam N Pham, Shankar Rai, Barclay T Stewart, Kiran K Nakarmi

**Affiliations:** University of Washington, Seattle, Washington; Kirtipur Hospital, Kathmandu, Bagmati; University of Washington, Seattle, Washington; Kirtipur Hospital, Phect-Nepal, Kathmandu, Bagmati; Kirtipur Hospital, Phect-Nepal, Kathmandu, Bagmati; Kirtipur Hospital, Phect-Nepal, Kathmandu, Bagmati; Kirtipur Hospital, Phect-Nepal, Kathmandu, Bagmati; University of Washington, Seattle, Washington; Kirtipur Hospital, Phect-Nepal, Kathmandu, Bagmati; University of Washington, Seattle, Washington; Kirtipur Hospital, Phect-Nepal, Kathmandu, Bagmati

## Abstract

**Introduction:**

Protocolized burn resuscitation algorithms with hourly, closed loop feedback, have reduced instances of over- and under-resuscitation and improved outcomes in high income countries. However, a “know-do” gap exists as this practice has yet to be adopted in many low- and middle-income countries (LMIC). We aimed to describe the change management process of the development and implementation of a contextually driven protocolized burn resuscitation bundle at a tertiary burn center in an LMIC using an implementation science framework.

**Methods:**

We applied strategies from the Expert Recommendations for Implementing Change (ERIC) for the design and implementation of a burn resuscitation bundle at a major burn center in an LMIC, over a 9-month period. Semi-structured focus group discussions (FGD) were conducted with stakeholders to understand facilitators and barriers to developing and using the protocol, with iterative feedback used to inform and adjust the protocol and documentation tools. Responses were analyzed using content analysis and particularly unique and useful responses were highlighted.

**Results:**

Stakeholders identified resource constraint-related concerns about the feasibility of an *hourly* IV resuscitation protocol and reached consensus on performing *2-hourly* assessments and fluid adjustments. Corresponding documentation tools were developed and iteratively adjusted. Several initial barriers to adoption and institutionalization were encountered. ERIC strategies used to promote intervention uptake included simplification and visualization of the protocol, identification of a project champion, development of educational materials for multiple cadres (e.g., nurses, physicians, health assistants), use of chain of command to enable change and accountability, utilizing institutional branding and ultimately obtaining endorsement by the center’s leadership (Table 1). Post-implementation FGD with stakeholders revealed high levels of acceptance, utilization and adherence of the protocol bundle, with occasional opportunities for improvement identified in protocol completeness and accuracy.

**Conclusions:**

Adoption of change in clinical resuscitation practice in a resource-constrained setting required a contextually driven, multi-faceted approach led by a team of change champions and leaders. The ERIC framework allowed for an iterative approach to prioritize stakeholder engagement and feedback, in order to implement a protocolized IV resuscitation bundle in a LMIC.

